# Measuring depression with CES-D in Chinese patients with type 2 diabetes: the validity and its comparison to PHQ-9

**DOI:** 10.1186/s12888-015-0580-0

**Published:** 2015-08-18

**Authors:** Yuying Zhang, Rose Z W Ting, Marco H B Lam, Siu-Ping Lam, Roseanne O. Yeung, Hairong Nan, Risa Ozaki, Andrea O Y Luk, Alice P S Kong, Yun-Kwok Wing, Norman Sartorius, Juliana C N Chan

**Affiliations:** 1 Department of Medicine and Therapeutics, Prince of Wales Hospital, The Chinese University of Hong Kong, Hong Kong SAR, China; 2 Department of Psychiatry, The Chinese University of Hong Kong, Hong Kong SAR, China; 3 Hong Kong Institute of Diabetes and Obesity, Hong Kong SAR, China; 4 Asia Diabetes Foundation, The Chinese University of Hong Kong, Hong Kong SAR, China; 5 The Association for the Improvement of Mental Health Programmes, Geneva, Switzerland

## Abstract

**Background:**

The validity of the 20-item Center for Epidemiological Studies Depression (CES-D) scale for depression screening in Hong Kong Chinese patients with type 2 diabetes remains unknown. We aimed to validate CES-D, compare its psychometric properties with the 9-item Patient Health Questionnaire (PHQ-9), and explore whether one of the two is more suitable for depression screening in Chinese patients with type 2 diabetes.

**Methods:**

Between June 2010 and July 2011, 545 consecutive Chinese patients with type 2 diabetes who underwent structured comprehensive assessments completed the CES-D and PHQ-9. Forty patients were retested within 2–4 weeks by telephone interview and 97 patients were randomly selected to undergo the Mini International Neuropsychiatric Interview (MINI) by psychiatrists for clinical diagnosis of depression.

**Results:**

The internal consistency (Cronbach’s α) of CES-D was 0.85, with a test-retest correlation coefficient of 0.64. The area under the curve for CES-D compared to the clinical diagnosis of major depression was 0.85. A cut-off score of ≥21 for CES-D provided the optimal balance between sensitivity (78.3 %) and specificity (74.3 %) and identified 17.8 % (*n* = 97) of patients with depression. CES-D and PHQ-9 showed moderate agreement in depression screening (Cohen’s Kappa: 0.45). Compared to non-depressed patients, those who screened positive by PHQ-9 had a higher HbA_1c_ whereas the glycemic differences were not significant when using CES-D.

**Conclusion:**

The CES-D is a valid screening tool for depression in Chinese type 2 diabetic patients although the PHQ-9 was more discriminative in identifying those with suboptimal glycemic control.

## Background

Depression and type 2 diabetes are complex diseases with rising prevalence [1, 2]. These two chronic conditions frequently coexist resulting in increased risk of morbidity and mortality with major negative implications on the individuals, families and society [3, 4]. International diabetes guidelines now recommend screening for psychosocial problems including depression, especially when self-management is poor [5, 6]. The 20-item Center for Epidemiological Studies Depression (CES-D) scale and the 9-item Patient Health Questionnaire (PHQ-9) are two most widely used self-administered instruments for depression screening [7, 8]. Originally developed for a general population in a Western setting, both instruments have been validated in other populations including American and Hong Kong Chinese community-dwelling individuals [9–13].

There is emerging evidence suggesting that ethnicity, culture, and acculturation may lead to response bias in these instruments [13–15]. We previously reported the validity of PHQ-9 for depression screening in Hong Kong Chinese patients with type 2 diabetes and reported a lower cutoff point (≥7) for significant depressive symptoms than the conventional one (≥10) which was first validated in primary care settings and obstetrics-gynecology clinics in U.S. [13, 16]. However, there is a paucity of data on the performance of CES-D in Chinese patients with type 2 diabetes. In this study, we aimed to validate CES-D and compare its psychometric properties with PHQ-9 in community-dwelling Chinese patients with type 2 diabetes in Hong Kong.

## Methods

### Subjects and setting

The study design and patient recruitment have been described previously [16]. In brief, 601 Chinese outpatients with type 2 diabetes aged 25–75 years were recruited consecutively from a hospital-based (Prince of Wales Hospital) and a community-based (Yao Chung Kit Diabetes Assessment Centre) diabetes center between June 2010 and July 2011. All patients underwent a 4-hour diabetes complication assessment using a structured protocol provided by the Joint Asia Diabetes Evaluation Program [17–20]. They were also invited to complete a set of questionnaires to assess their psychological well-being. Significant medical and psychiatric history, social history, family history of diabetes, and medication records were documented. Urine and blood samples were collected after overnight fast for plasma glucose, glycated hemoglobin (HbA_1c_), total cholesterol, low density-lipoprotein cholesterol (LDL-C), high density-lipoprotein cholesterol (HDL-C), triglycerides, renal function, and urinary albumin-to-creatinine ratio (ACR). This study was approved by the ethics committee of The Chinese University of Hong Kong, and all patients gave informed consent.

### Psychological assessment

Symptoms of depression were assessed by the CES-D and PHQ-9 questionnaires. The CES-D scale is a 20-item self-reported instrument developed by Radloff in 1977 [7]. It measures the frequency of common depressive symptoms over the past week. Each item is scored from 0 (rarely or none of the time, less than one day) to 3 (all of the time, 5–7 days). The four positively stated items (item 4, I felt that I was just as good as other people; item 8, I felt hopeful about the future; item 12, I was happy; item 16, I enjoyed life) are reverse-coded for calculating the total score which ranges from 0 to 60. The cut-off value of ≥16 has been widely used to define clinically meaningful depressive symptoms [7, 21]. It was reported to have 96.8 % sensitivity and 67.6 % specificity for clinical depression in Chinese type 2 diabetic patients attending a diabetes centre in Singapore [22]. The PHQ-9 focuses on the frequency of occurrence of 9 depressive symptoms derived from DSM-IV diagnostic criteria over the past two weeks [8]. Each item is scored from 0 (not at all) to 3 (nearly every day), with a total score ranging from 0 to 27. A cutoff value of 10 has been widely used to define probable depression, with 88 % sensitivity and 88 % specificity in the original validation study with majority of participants being Caucasians [8]. In our previous criterion validation in the same group of 99 patients, we identified the optimal value of 7 with 82.6 % sensitivity and 73.7 % specificity [16].

The process of this study has been described previously [16]. Briefly, 40 patients were randomly selected for CES-D and PHQ-9 retest within 2–4 weeks by telephone survey. Another randomly selected subset of patients was referred for assessment by psychiatrists (Dr Marco Lam and Dr Siu-ping Lam) using the Chinese version of Mini International Neuropsychiatric Interview (MINI version 6.0), a short structured diagnostic interview that has been validated and is widely accepted for diagnosing major depression in a research setting [23, 24]. Due to manpower issue, patient was assessed by one psychiatrist only and we were not able to calculate the inter-rater reliability. However the two psychiatrists received training for MINI together. Pilot interview was conducted in three patients and it showed 100 % agreement in the MINI diagnosis for depression.

### Statistical analyses

All analysis was performed using the Statistical Package for Social Sciences (SPSS version 20.0, IBM). Data were expressed as mean ± SD, median (interquartile range) or number (%), as appropriate. The Student’s t-test, Mann–Whitney U test and Chi-square tests were used for group comparisons. Cronbach’s α was calculated to evaluate the internal consistency. Pearson correlation coefficients were used to measure test-retest correlation and concurrent validity of the PHQ-9 and CES-D as appropriate. Item discrimination was tested by corrected item-total correlation using the Pearson product–moment correlation formula, which has been incorporated into SPSS Reliability analysis. Exploratory factor analysis (EFA) with eigenvalue >1 criteria was performed to establish the construct validity. An oblique (Promax) rotation was used in the EFA based on the assumption that the CES-D would have correlated factors. The response agreement between PHQ-9 and CES-D was evaluated with Cohen’s kappa. Receiver Operator Characteristic (ROC) analysis was used to determine the diagnostic performance and optimal cutoff score for screening major depression against MINI-based clinical diagnosis. A*P* value < 0.05 (2-tailed) was considered significant.

## Results

### Characteristics of study participants

A total of 545 participants (mean age 54.6 ± 9.5 years, median[IQR] disease duration 6 [3–11] years, 41.3 % were female) fulfilled the inclusion criteria and had complete CES-D and PHQ-9 data for analysis, among them 97 underwent clinical interview by psychiatrists. Their characteristics were presented in Table 1. Overall, 4.8 % (*n* = 26) of patients had a history of clinical diagnosed psychiatric disorder, mostly consisting of depression (*n* = 18), and 8.2 % (*n* = 44) of patients were concurrently treated with psychotropic medications. 28 (5.1 %) had suicide ideation in the past 2 weeks based on the 9th item of PHQ-9. Of them, 22 (4.0 %), 5 (0.9 %) and 1 (0.2 %) reported suicide ideation for a few days, more than half the days and nearly every day, respectively.

**Table 1:** Demographic and clinical characteristics of Chinese type 2 diabetic patients categorised by validated cutoff values of PHQ-9 and CES-D

	Total	CES-D <21	CES-D ≥21	*p* value	PHQ-9 < 7	PHQ-9 ≥ 7	*P* value
Number	545	448	97 (17.8)		449	96 (17.6)	
CES-D score	13.0 ± 8.6	9.9 ± 5.3	27.4 ± 5.6	<0.001	11.0 ± 6.9	22.3 ± 9.5	<0.001
PHQ-9 score	3.5 ± 4.4	2.3 ± 2.7	8.8 ± 6.2	<0.001	1.8± 1.8	11.2± 4.6	<0.001
Age (years)	54.6 ± 9.5	54.7 ± 9.7	54.4 ± 8.6	0.807	54.4 ± 9.4	55.4 ± 9.9	0.339
Women	225 (41.3)	173 (38.6)	52 (53.6)	0.007	170 (37.9)	55 (57.3)	<0.001
Disease duration (years)	6.0 (3.0,11.0)	7.0 (3.0,10.3)	6.0 (3.0,13.0)	0.867	7.0 (4.0,10.8)	6.0 (2.0-13.0)	0.568
Education				0.555			0.073
Illiterate/primary school	142 (26.1)	117 (26.1)	25 (25.8)		113 (25.2)	29 (30.2)	
Middle school	275 (50.5)	222 (49.6)	53 (54.6)		222 (49.4)	53 (55.2)	
High school and above	128 (23.5)	109 (24.3)	19 (19.6)		114 (25.4)	14 (14.6)	
In employment	307 (56.3)	259 (57.8)	48 (19.5)	0.134	266 (59.2)	41 (42.7)	0.003
Current smoker	56 (10.3)	49 (11.0)	7 (7.2)	0.271	43 (9.6)	13 (13.5)	0.249
Exercise ≥3 times/week	262 (48.1)	216 (48.2)	46 (47.4)	0.887	217 (48.3)	45 (46.9)	0.796
SMBG ≥ weekly	278 (51.0)	234 (52.2)	44 (45.4)	0.22	232 (51.7)	46 (47.9)	0.504
Cardio-metabolic risk factors							
Body mass index (kg/m^2^)	26.1 ± 4.4	26.1 ± 4.3	26.0 ± 4.9	0.802	26.1 ± 4.4	26.0 ± 4.6	0.902
Systolic BP (mmHg)	130.6 ± 15.7	130.3 ± 15.5	132.3 ± 16.7	0.255	130.4 ± 15.6	131.7 ± 16.2	0.464
Diastolic BP (mmHg)	79.6 ± 10.0	79.3 ± 9.9	80.9 ± 10.5	0.158	79.5 ± 10.0	80.2 ± 10.2	0.510
Waist circumference, men (cm)	91.2 ± 10.5	91.1 ± 10.2	91.8 ± 12.1	0.713	91.3 ± 10.4	90.7 ± 11.3	0.742
Waist circumference, women (cm)	86.1 ± 11.1	85.8 ± 10.9	87.2 ± 11.7	0.421	85.8 ± 11.5	87.0 ± 10.0	0.475
HbA1c (%)	7.5 ± 1.4	7.5 ± 1.4	7.7 ± 1.6	0.238	7.4 ± 1.3	7.9 ± 1.9	0.045
HbA1c (mmol/L)	58.6 ± 15.7	58.3 ± 15.2	60.4 ± 18.0	0.238	57.8 ± 14.2	62.4 ± 21.2	0.045
Total cholesterol (mmol/L)	4.50 ± 0.88	4.48 ± 0.87	4.60 ± 0.91	0.247	4.52 ± 0.89	4.40 ± 0.84	0.200
HDL-C (mmol/L)	1.34 ± 0.37	1.33 ± 0.37	1.35 ± 0.36	0.632	1.35 ± 0.37	1.30 ± 0.37	0.223
LDL-C (mmol/L)	2.49 ± 0.71	2.48 ± 0.70	2.56 ± 0.75	0.284	2.51 ± 0.71	2.42 ± 0.72	0.324
eGFR (ml/min/1.73 m^2^)	109.0 ± 23.7	109.3 ± 23.4	108.1 ± 24.9	0.654	109.3 ± 23.8	108.0 ± 23.2	0.653
Spot urinary ACR	1.10 (0.48,4.02)	1.09 (0.48,3.90)	1.11 (0.51,4.56)	0.442	1.05 (0.44,3.90)	1.25 (0.62,5.03)	0.096
Hypertension	430 (78.9)	356 (79.5)	74 (76.3)	0.487	349 (77.7)	81 (84.4)	0.147
Dyslipidaemia	463 (85.3)	383 (85.9)	80 (82.5)	0.345	380 (85.0)	83 (86.5)	0.717
Albuminuria	166 (30.5)	136 (30.4)	30 (30.9)	0.912	135 (30.1)	31 (32.3)	0.667
Diabetes-related complications							
Cardiovascular disease	53 (9.7)	42 (9.4)	11 (11.3)	0.554	40 (8.9)	13 (13.5)	0.164
Retinopathy	112 (20.6)	91 (20.3)	21 (21.6)	0.786	95 (21.2)	17 (17.7)	0.448
Chronic kidney disease	10 (1.8)	8 (1.8)	2 (2.1)	0.695	8 (1.8)	2 (2.1)	0.689
Sensory neuropathy	13 (2.4)	7 (1.6)	6 (6.2)	0.016	7 (1.6)	6 (6.2)	0.015
Medication, target achievement							
Use of insulin	87 (16.0)	73 (16.3)	14 (14.4)	0.65	73 (16.3)	14 (14.6)	0.684
On OAD	492 (90.3)	402 (89.7)	90 (92.8)	0.358	402 (89.5)	90 (93.8)	0.206
Use of antihypertensive drugs	324 (59.4)	269 (60.0)	55 (56.7)	0.543	261 (58.1)	63 (65.6)	0.704
Use of lipid lowering drugs	265 (48.6)	223 (49.8)	42 (43.3)	0.247	208 (46.3)	57 (59.4)	0.02
Concurrent use of psychotropic drugs	44 (8.2)	16 (4.3)	28 (16.7)	<0.001	27 (6.1)	17 (17.7)	<0.001
HBA_1C_ < 7.0 % (53 mmol/L)	218 (40.0)	181 (40.4)	37 (38.1)	0.681	187 (41.6)	31 (32.3)	0.089
LDL-C < 2.6 mmol/L	312 (57.2)	255 (56.9)	57 (58.8)	0.739	252 (56.1)	60 (62.5)	0.252
BP < 130/80 mmHg	206 (37.8)	173 (38.6)	33 (34.0)	0.397	175 (39.0)	31 (32.3)	0.22

*PHQ-9* Patients Health Questionnaire-9, *CES-D* 20-item Center for Epidemiological Studies Depression, *SMBG* Self-monitoring of blood glucose, *BP* Blood pressure, *eGFR* estimated glomerular filtration rate, *ACR* albumin-to-creatinine ratio, *OAD* Oral antidiabetic drugs

Data are shown as mean ± SD, number (%) or median (interquartile range)

The definitions of risk factors and complications were as follows: hypertension = known high blood pressure with or without treatment and/or blood pressure ≥ 130/80 mmHg; dyslipidaemia = LDL-C ≥ 2.6mmol/L, HDL-C < 1.0 mmol/L, triglycerides ≥ 2.3 mmol/L, or on any lipid-lowering agents; albuminuria = sport urine albumin/creatinine ratio ≥ 2.5 mg/mmol in men or ≥ 3.5 mg/mmol in women; chronic kidney disease = eGFR <60 ml/min/1.73 m^2,^ cardiovascular disease = coronary heart disease, stroke, and/or peripheral vascular disease

### Psychometric properties of the CES-D

#### Internal reliability and item discrimination

The internal consistency (Cronbach’s α) of CES-D was 0.85, with test-retest correlation coefficient (*r*) of 0.64 (*P* < 0.001), similar to PHQ-9 (α = 0.87, *r* = 0.70, *P* < 0.001) as shown in our previous study [16]. After removal of the four positive affective items in the CES-D the internal consistency of CES-D questionnaire increased to 0.91. The corrected item-total correlations for individual CES-D items ranged from 0.17 (item 4: feeling good) to 0.66 (item 6: depressed) (Table 2), lower for the four positive items than other items. The corrected item-total correlations for individual PHQ-9 items ranged from 0.48 to 0.68 [16].

**Table 2:** Item scores, internal reliability, factor loadings and inter-factor correlations of CES-D

	Score		Internal reliability (α = 0.852)		Factor loadings^b^			
	Mean	SD	Corrected item-total correlation	α if item deleted^a^	Factor 1	Factor 2	Factor 3	Factor 4
1. I was bothered by things that usually don’t bother me.	0.72	0.84	0.56			0.73		
2. I did not feel like eating; my appetite was poor.	0.36	0.68	0.31			0.65		
3. I felt that I could not shake off the blues even with help from my family or friends.	0.40	0.71	0.62		0.69			
4. I felt I was just as good as other people.	1.65	1.19	0.17	0.86			0.77	
5. I had trouble keeping my mind on what I was doing	0.49	0.70	0.50			0.69		
6. I felt depressed.	0.47	0.72	0.66		0.77			
7. I felt that everything I did was an effort.	0.48	0.72	0.48			0.65		
8. I felt hopeful about the future	1.50	1.20	0.21	0.86			0.80	
9. I thought my life had been a failure.	0.44	0.77	0.56		0.67			
10. I felt fearful	0.44	0.74	0.62		0.76			
11. My sleep was restless.	0.66	0.85	0.46			0.64		
12. I was happy.	1.39	1.16	0.33	0.85			0.87	
13. I talked less than usual.	0.61	0.84	0.51			0.62		
14. I felt lonely.	0.45	0.76	0.63		0.73			
15. People were unfriendly	0.39	0.66	0.51					0.88
16. I enjoyed life.	1.36	1.19	0.30	0.86			0.88	
17. I had crying spells.	0.35	0.68	0.46		0.74			
18. I felt sad	0.39	0.70	0.64		0.87			
19. I felt that people dislike me	0.33	0.62	0.43					0.88
20. I could not get “going.”	0.14	0.48	0.51		0.65			
Factor score					3.1 ± 4.2	3.3 ± 3.2	5.9 ± 3.9	0.7 ± 1.2
Inter-factor correlation								
Factor 1					1.00	0.75*	0.04	0.53*
Factor 2						1.00	−0.05	0.49*
Factor 3							1.00	−0.02

^a^Only those with increased Cronbach’s α if item deleted were shown in the table

^b^Factor 1: Depressed affect; factor 2: somatic symptoms; factor 3: positive affect; factor 4: Interpersonal problems

*P for Pearson correlation (*r*) <0.0001. The correlations between positive affect and other subscales were not significant

### Construct validity and item scores of CES-D

**Fig. 1:**
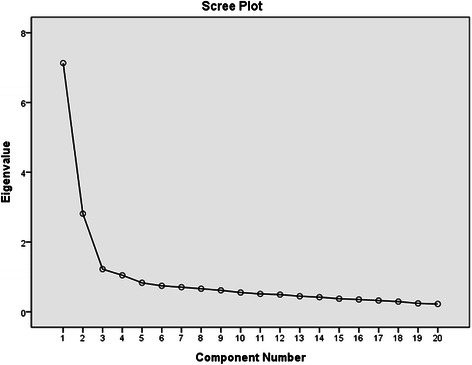
The scree plot for CES-D exploratory factor analysis

The Kaiser-Meyer-Olkin test of sampling adequacy was 0.91 and Bartlett’s test of sphericity was significant (*X*^2^ = 5042.6, *P* < 0.001). EFA using Promax rotation procedure yielded a four-factor structure for the CES-D according to the “eigenvalue >1” rule: 1) depressed affect, 2) somatic symptoms, 3) positive affect, and 4) interpersonal problems. The scree plot was shown as Fig. 1. This four-factor model accounted for 61.1 % of the scale variance, with factor loadings ranging from 0.62 to 0.88 (Table 2).

The mean CES-D total score was 13.0 ± 8.6 (median12.0, IQR 7.0-17.0), with individual item scores ranging from 0.14 to 1.65. The four positive affect items scored much higher than the other items, with a mean factor score of 5.9 ± 3.9, accounting for 50 % of the total CES-D score. The positive affect factor did not correlate with the somatic symptoms (*r* = −0.05,*P* = 0.275), depressed affect (*r* = 0.04, *P* = 0.414), or interpersonal problems (*r* = −0.02, *P* = 0.703); the latter three factors were significantly inter-correlated, with correlation coefficients ranging from 0.49 to 0.75 (all *P* < 0.001) (Table 2)

### Diagnostic validity

**Fig. 2:**
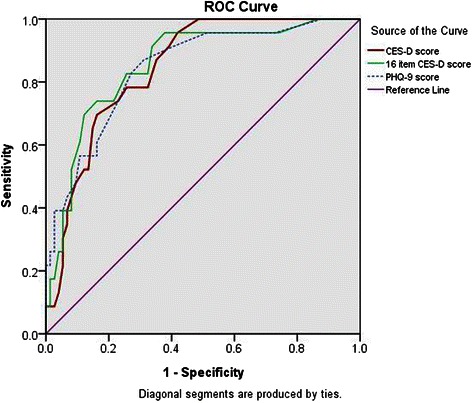
Receiver operating characteristic (ROC) curve analysis of CES-D and PHQ-9 versus clinical interview by MINI in 97 Chinese patients with type 2 diabetes

Among the 97 patients who were interviewed by two psychiatrists, 23 patients had a clinical diagnosis of current major depressive episode as measured by the MINI. The area under the curve (AUC) upon ROC analysis was 0.85(95%CI: 0.77-0.92) (Fig. 2). The standard cut-off score of ≥16 for CES-D had an excellent sensitivity (91.3 %) but low specificity (60.8 %) compared to the MINI diagnosis of major depressive episode. As shown in Table 3, a cut-off score of ≥21 on CES-D yielded an optimal balance between sensitivity (78.3 %) and specificity (74.3 %), with positive predictive value (PPV) of 48.6 %, and negative predictive value (NPV) of 91.7 %, similar to the PHQ-9 score ≥ 7. Furthermore, the removal of the four positive items slightly improved the diagnostic performance (Cronbach’s α 0.91, AUC 0.85 [0.77-0.94]), where the optimal cutoff value of 14 yielded a sensitivity of 82.6 % and specificity of 74.3 % (PPV 50.0 %, NPV 93.2 %).

**Table 3:** Diagnostic performance for detection of major depressive disorder using MINI clinical interview as reference (*n* = 97)

	Cutoff value	Sensitivity	Specificity	LR+	LR-	PPV	NPV
20-item CES-D							
	≥16	91.3 %	60.8 %	2.33	6.99	42.0 %	95.7 %
	≥17	87.0 %	64.9 %	2.47	4.97	43.5 %	94.1 %
	≥18	78.3 %	67.6 %	2.41	3.11	42.9 %	90.9 %
	≥19	78.3 %	71.6 %	2.76	3.29	46.2 %	91.4 %
	≥20	78.3 %	71.6 %	2.76	3.29	46.2 %	91.4 %
	≥21	78.3 %	74.3 %	3.05	3.42	48.6 %	91.7 %
	≥22	73.9 %	77.0 %	3.22	2.95	50.0 %	90.5 %
16-item CES-D							
	≥13	82.6 %	73.0 %	3.06	4.20	48.7 %	93.1 %
	≥14	82.6 %	74.3 %	3.22	4.27	50.0 %	93.2 %
	≥15	73.9 %	78.4 %	3.42	3.01	51.5 %	90.6 %
PHQ-9							
	≥6	87.0 %	68.9 %	2.80	5.28	46.5 %	94.4 %
	≥7	82.6 %	73.0 %	3.06	4.20	48.7 %	93.1 %
	≥8	73.9 %	77.0 %	3.22	2.95	50.0 %	90.5 %
	≥9	60.9 %	83.8 %	3.75	2.14	53.8 %	87.3 %
	≥10	56.5 %	83.8 %	3.49	1.93	52.0 %	86.1 %

*LR+* Positive likelihood ratio, *LR-* Negative likelihood ratio, *PPV* Positive predictive ratio, *NPV* Negative predictive ratio

### Comparison of CES-D and PHQ-9

**Fig. 3:**
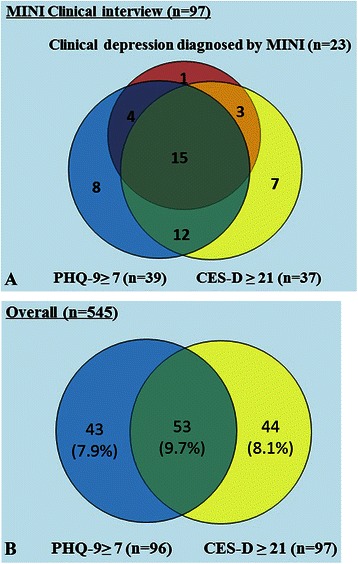
Venn diagram. **a** for those with MINI interview; **b** among all the patients

If the conventional cut-off points (score of ≥16 for CES-D and ≥10 for PHQ-9) were adopted, 31.0 % and 9.0 % of patients were respectively identified to have depression by CES-D and PHQ-9, with fair chance-corrected agreement between the two instruments (Cohen’s kappa: 0.32). However if we used a score of 21 as the cut-off for CES-D, 17.8 % of patients were identified to have possible depression, similar to the depression prevalence reported in the same group of patients using the PHQ-9 ≥ 7 [16], with moderate chance-corrected agreement between the two instruments (Cohen’s kappa: 0.45). The overlap of depressive symptom screen positivity is illustrated in a Venn diagram (Fig. 3), where just over a third of the 545 patients were captured by both PHQ-9 ≥ 7 and CES-D ≥21.

Overall, the demographic and clinical patterns of depression versus non-depression were similar using either PHQ-9 or CES-D as the screening instrument. However, patients with depression by PHQ-9 had significantly higher HbA_1c_ level while there was no such difference between the depressed and non-depressed groups defined by the 20-item CES-D (Table 1), nor did by the 16-item CES-D (7.6 ± 1.7 *vs*. 7.5 ± 1.4 % [59.2 ± 18.4 *vs*. 58.6 ± 15.2 mmol/l], *P* = 0.734). Similarly, PHQ-9 score as a continuous value was positively associated with high HbA_1c_ level (*r* = 0.12, *P* = 0.004) but not for CES-D score (*r* = 0.03, *P* = 0.423). Patients positive for both CES-D (≥21) and PHQ-9 (≥7) had similar HbA_1c_ to those positive only for PHQ-9 (7.9 ± 1.9 *vs*. 7.9 ± 2.1 % [62.5 ± 20.6 *vs*. 62.4 ± 22.5 mmol/l], *P* = 1.000) but higher HbA_1c_ than those positive only for CES-D although it did not reach statistical significance (7.9 ± 1.9 *vs*. 7.4 ± 1.3 % [62.5 ± 20.6 *vs*. 57.8 ± 14.5 mmol/l], *P* = 0.206). Moreover, using PHQ-9 ≥ 7 as the cutoff value, patients with depression were more likely to use lipid lowering drugs. Such difference was not detected if we used CES-D ≥21 to define depression.

## Discussion

To our best knowledge, this is the first study to validate CES-D and systematically compare it with the PHQ-9 for depression screening in Chinese patients with type 2 diabetes. The internal consistency, test-retest reliability, and diagnostic performance of CES-D was comparable with that of the PHQ-9, which had been validated in the same population [16].

### Factor structure of CES-D

The four-factor structure of CES-D was similar to the original one proposed by Radloff [7], except that item 20 (“get going”) loaded in the depressed affect factor but not the somatic symptom factor in our Chinese patients with type 2 diabetes. These subtle variations in factor structure might be due to racial/ethnic or culture differences [25, 26]. In a meta-analysis on CES-D factor structure, people from different cultures might conceptualize depressive symptoms in different ways. Besides, the analytic methods used to load various factors, e.g. confirmatory factor analysis (CFA) versus EFA, could also influence the results of CES-D factor structure [25]. In another study involving 138 Hong Kong Chinese married couples (aged 22–70 years) which used CFA to validate the CES-D, the authors reported a 2-factor model (depression and interpersonal problems) [11] compared to the 4-factor model in our study.

In this study, the positive affect factor did not correlate with the other three factors (inter-factor correlation ranged from −0.05 to 0.04), contrary to many other studies in United States (ranged from 0.31 to 0.85) [27–29]. Traditional Chinese and Oriental culture emphasizes modesty, silence, stoicism, and emotional restraint. These beliefs might influence our patients not to endorse positively-stated items in the CES-D (e.g. “I was happy”, the score was reversed during calculation) despite having other negative symptoms, leading to an elevated score on positive affect problems. Our results are consistent with other studies in Chinese subjects [14, 30]. In a study of 168 community-dwelling American Chinese women, native Chinese speakers or Chinese immigrants were 50 % less likely to endorse the four positive items than English speakers or subjects born in United States, albeit having similar mean scores for the other 16 items [14]. This discrepancy has also been reported in other studies probably due to culture influences [30, 31]. American Koreans who were less acculturated to American views were less likely to endorse positive CES-D items than the more acculturated ones [32]. Compared to Americans, Japanese had spuriously lower ratings of positive items whereas the scores for the negative items were comparable between the two groups [30]. Taken together, these cultural or ethnic factors seemed to affect responses to the four positive affect items which might compromise the validity of CES-D. In support of this, the performance of CES-D improved upon exclusion of these 4 positive affect items suggesting that the 16 item CES-D is a better screening tool for depression than the 20 item CES-D, at least in Chinese subjects.

### Higher cut-off point of CES-D in Chinese

Our results suggested an optimal cutoff value of 21, which is higher than the widely used cutoff value of 16 (sensitivity 91.3 %, specificity 60.8 %). Different study designs, settings and populations may contribute to differences in the performance of different cutoff values [33]. Consistently, in studies involving Chinese subjects, the latter tended to have higher CES-D cut-off points in depression screening than Caucasian populations [12, 22, 34]. For example, a CES-D validation study in Hong Kong involving 398 elderly individuals reported an optimal cutoff value of 22 (75 % sensitivity and 51 % specificity), while the conventional cutoff value of 16 had high sensitivity (92 %) but poor specificity (30 %) in detecting depression [12]. In another CES-D validation study in Singaporean Chinese adults using the Schedule for Clinical Assessment in Neuropsychiatry (SCAN) as criterion, the cutoff value of 16 had specificity of 67.6 % only, although the sensitivity was high (96.8 %) [22].

### Comparison of CES-D to PHQ-9

In line with other studies [35, 36], our findings showed that both PHQ-9 and CES-D showed similar psychometric performances with respect to the internal consistency, test-retest reliability, and diagnostic validity against MINI-based diagnostic interview. Using the standard cutoff point (≥16), CES-D identified more than 30 % of patients with possible depression, much higher than using the standard cutoff point ≥10 of PHQ-9 (9.0 %, Cohen’s Kappa = 0.32). This finding is consistent with a recent study in systemic sclerosis where Milette and colleagues compared the PHQ-9 to CES-D in 566 patients with systemic sclerosis and found CES-D ≥ 16 identified 34.3 % patients with possible depression, while PHQ-9 ≥ 10 identified 20.7 % (Cohen’s Kappa = 0.49), suggesting that PHQ 9 is more specifically indicative of depression while the CES-D is capturing some of the depression and some of the other emotions present in serious illness.

Compared to conventional cutoff, the CES-D had a higher cutoff value while PHQ-9 had a lower cutoff value. This disparity might be partly explained by the content differences of the two tools. The PHQ-9 is constructed on the basis of the DSM-IV diagnostic criteria for clinical depression; while the CES-D measures depressive symptomatology with emphasis on the affective component and depressed mood [7]. Besides, the PHQ-9 asks about the frequency of depressive symptom in the past two weeks, while the CES-D asks about the frequency of symptoms in the past one week. The shorter duration covered by the CES-D may have captured short-term symptoms including acute hassles and stressors which might not reflect true depression. Besides, since Chinese people tend to give negative response to positive affect items, this may also lead to a higher score of CES-D in our study population.

While the use of PHQ-9 (≥7 or ≥ 10) identified patients with significant depressive symptoms which were associated with poor glycemic control and increased use of lipid-lowering drugs, such association was not found in patients with depressive symptoms detected by CES-D, suggesting that CES-D might identify patients with slightly different profile than those identified by PHQ-9. When evaluating other studies that have examined the association between depression and glycemic control, findings have been mixed where some studies have found an association between depressive symptoms and poor glycemic control whereas others have not [37, 38]. Our results raise the possibility that this inconsistency might be in part due to the different tools used in capturing depressive symptoms. Here, negative emotions can be heterogeneous and complex with different combinations of symptoms which may have common but also distinct biological pathways. This complexity may also explain the inconsistency regarding the associations between depression and glycemic control.

Although theoretically, the CES-D and the PHQ-9 may complement one another to detect depression and negative emotions, this may be not feasible in real-world practice given increased time of testing and redundancy of the items. However, since the two tools cover different time frame (1 week versus 2 weeks) with slightly different attributes (eg. interpersonal problems in CES-D and suicidal tendency in PHQ-9) and complementary aspects as revealed by the Venn diagram, it might be useful to explore the possibility of selecting items from both tools to generate a better depression screening algorithm in future studies.

Both PHQ-9 and CES-D had different cutoff values with PHQ-9 being a better tool than the CES-D in identifying Chinese type 2 diabetic patients with both depression and poor glycemic control. These results highlighted the importance of validating screening tools in local settings. The differences in associations of depression with glycemic control between the two instruments also support the syndromic nature of depression due to possible subphenotypes and aetiologies with variable responses to different screening tools. In this study, we also observed a high suicide risk in these patients that 5 % had suicide ideation using the PHQ-9. Consistently, a study in Italy found more severe suicide ideation in patients with diabetes and its severity was closely associated with older age, polytherapy and lower self-efficacy [39]. Therefore, these results warrant depression screening and subsequent emotional support for patients with diabetes.

### Limitations

Although the study population comprised of self-referred patients and those referred by family clinics, the majority of them were attending hospital-based specialist out-patient clinics, who might have more severe disease, longer disease duration, multiple co-morbidities, and more complicated drug regimens than the typical outpatient community-based patients. Thus, our cohort might not fairly represent the general Hong Kong Chinese population with type 2 diabetes. Furthermore, this study was performed in Hong Kong, which has a different health care system and different cultural nuances than that of Mainland China, so caution must be taken when making generalizations to the wider Chinese population. Finally, only a subset of our cohort had a diagnostic interview as the criteria validation measure, so further studies with larger sample sizes are required to confirm these findings.

## Conclusions

In summary, the CES-D is a validated tool for detecting major depression in Chinese patients with type 2 diabetes. The improvement in performance after excluding items on positive affect might reflect cultural differences. Between CES-D and PHQ-9, the latter is a preferred screening tool due to its longer coverage period, fewer items associated less administering time, and ability to identify depressed patients with poor metabolic control. The different cutoff values in our population also emphasize the importance of calibrating these tools in different patient groups and settings.
